# Synchronous Extraction, Antioxidant Activity Evaluation, and Composition Analysis of Carbohydrates and Polyphenols Present in Artichoke Bud

**DOI:** 10.3390/molecules27248962

**Published:** 2022-12-16

**Authors:** Xiao Lin, Xian-Kun Lu, Kai-Hao Zhu, Xin-Yang Jiang, Jiong-Chao Chen, Pei-Zheng Yan, Dong-Sheng Zhao

**Affiliations:** 1 College of Pharmacy, Shandong University of Traditional Chinese Medicine, Jinan 250355, China; 2 School of Pharmaceutical Engineering, Shenyang Pharmaceutical University, Shenyang 110016, China

**Keywords:** artichoke bud, synchronous extraction, aqueous two-phase system, dual-response surface model, antioxidant activity, carbohydrates, polyphenols

## Abstract

This study investigated the optimization of ultrasonic-assisted aqueous two-phase synchronous extraction of carbohydrates and polyphenols present in artichoke bud, evaluated their antioxidant activities in vitro, and analyzed the composition of carbohydrates and polyphenols by high-performance liquid chromatography (HPLC). The powder mass, ultrasonic time, ammonium sulfate concentration, and alcohol–water ratio were considered the influencing factors based on the single-factor experiment results, and a dual-response surface model was designed to optimize the synchronous extraction process to extract carbohydrates and polyphenols. The antioxidant activity was evaluated by measuring the scavenging capacity of ABTS^+^· and DPPH· and the reducing capacity of Fe^3+^. The optimal process conditions in this study were as follows: the powder mass of 1.4 g, ammonium sulfate concentration of 0.34 g/mL, alcohol–water ratio of 0.4, and ultrasonic time of 43 min. The polyphenol content in artichoke bud was 5.32 ± 0.13 mg/g, and the polysaccharide content was 74.78 ± 0.11 mg/g. An experiment on in vitro antioxidant activity showed that both carbohydrates and polyphenols had strong antioxidant activities, and the antioxidant activity of polyphenols was stronger than that of carbohydrates. The HPLC analysis revealed that the carbohydrates in artichoke bud were mannose, rhamnose, glucuronic acid, galacturonic acid, glucose, galactose, and arabinose, and the molar ratio was 10.77:25.22:2.37:15.74:125.39:48.62:34.70. The polyphenols comprised chlorogenic acid, 4-dicaffeoylquinic acid, caffeic acid, 1,3-dicaffeoylqunic acid, isochlorogenic acid B, isochlorogenic acid A, cynarin, and isochlorogenic acid C, and the contents were 0.503, 0.029, 0.022, 0.017, 0.008, 0.162, 1.621, 0.030 mg/g, respectively. This study also showed that the carbohydrates and polyphenols in artichoke bud could be important natural antioxidants, and the composition analysis of HPLC provided directions for their future research. Carbohydrates and polyphenols in artichoke buds can be separated and enriched using the optimized process technology, and it is an effective means of extracting ingredients from plants.

## 1. Introduction

Artichoke (*Cynara scolymus* L.), also known as chrysanthemum thistle, vegetable thistle, and so forth, is a perennial herb of the genus *Cynara* of the family Asteraceae [1]. This herb is native to the western and central Mediterranean regions [2] and is also cultivated in the Shanghai, Yunnan, and Zhejiang provinces of China. Nowadays, artichoke is widely used in pharmaceutical [3], food [4], and other industries. Modern pharmacological studies have shown that artichoke and its chemical components have various biological activities, such as antioxidant, antibacterial, antitumor, hepatoprotective, and cholesterol-lowering activities [1,5,6,7,8]. Artichoke not only has significant health benefits, it is also rich in a variety of functional components, such as flavonoids, carbohydrates, polyphenols, and terpenoids [3,9,10]. Besides its applications as a functional food and its bioactive ingredient sources, artichoke is recognized as a sustainable feedstock for biofuel production.

Reactive oxygen radicals are produced by the oxidative respiration metabolism of cells and have high reactivity because their outermost orbitals have unpaired electrons [11,12]. The activity of antioxidant enzymes in a body gradually decreases with increasing age, resulting in an accumulation of reactive oxygen radicals produced by cellular metabolism in the body. The accumulation of reactive oxygen radicals in the body leads to lipid peroxidation reactions [13,14], which can cause cell damage or even death. The body is prone to Alzheimer’s disease, Parkinson’s disease, diabetes, arthritis, cardiovascular diseases, tumors, and other diseases [15,16,17,18,19]. The research and development of antioxidants have become significant nowadays with experts trying to find ways to reduce damage caused by the presence of excessive reactive oxygen radicals in the human body, and natural drugs are gaining attention for their excellent antioxidant properties. Carbohydrates and polyphenolic compounds are the main natural components in artichokes with significant antioxidant activity [20,21], which is why they have become a significant topic of research for their use in pharmaceuticals, functional foods, and bioactive ingredient sources.

Carbohydrates or polyphenols are mainly extracted using the traditional solvent extraction method [22,23], enzymatic method [24], microwave method [25,26], ultrasonic method [25], and so forth. Although the traditional solvent extraction method is easy to operate, it is time-consuming and has low extraction efficiency [23]; the enzymatic method affects the extraction efficiency because of the easy inactivation of enzymes [27]; and the ultrasonic method uses cavitation and mechanical and thermal effects to significantly increase the extraction efficiency of active ingredients [28]. The dual-phase aqueous system can use environmentally friendly phase-forming components, such as ethanol and inorganic salts and can be rapidly phase-formed at room temperature, depending on the different partition coefficients of different substances in the two phases, thus enabling the rapid partitioning of the extracted substances in the two phases of this system [29,30,31]. The dual-phase aqueous extraction technique compared with the conventional extraction techniques used for the simultaneous extraction of active plant ingredients can reduce the associated energy consumption and has the advantages of high extraction efficiency, mild conditions, a nontoxic and environmentally-friendly nature, and simple operation [32,33]. Considering these advantages, the ultrasonic-assisted aqueous two-phase synchronous extraction method was used to extract carbohydrates and polyphenols out of artichoke bud.

The research on the extraction of the active components of artichoke currently focuses only on optimizing the extraction process targeting a single component (polyphenols) as an index [34,35]. In the absence of a multiple indicator extraction optimization process, the extraction of other active substances in artichoke cannot be performed, limiting the efficient development and utilization of artichoke resources. In this study, an ultrasonic-assisted ethanol–ammonium sulfate aqueous two-phase system was used to synchronously extract carbohydrates and polyphenols from artichoke bud. The extraction process was optimized using a dual-response surface model, and the composition and antioxidant activity in vitro were analyzed. It provided a theoretical basis for the in-depth study of the bioactive components of artichoke and also provided scientific guidance for the comprehensive development and utilization of artichoke.

## 2. Results and Discussion

### 2.1. Effects of the Investigated Factors on the Carbohydrate and Polyphenol Contents

#### 2.1.1. Effects of Ultrasonic Powers on the Carbohydrate and Polyphenol Contents in Artichoke Bud

When the ultrasonic power was in the range of 300–350 W, the extraction rate of carbohydrates from artichoke bud increased significantly (*p* < 0.05), but when the ultrasonic power continued to increase, the carbohydrates content decreased significantly (*p* < 0.05) (Figure S1A). Within this range, the variation in extraction trends of polyphenols and carbohydrates was roughly the same, but not as significant as that of carbohydrates. It was mainly because the cavitation effect of ultrasound could effectively dissolve carbohydrates and polyphenols present in artichoke bud, but the powerful ultrasonic power destroys the cell wall, causing the dissolved polyphenols to interact with carbohydrates to form insoluble aggregates, resulting in the reduction of content [36,37]. Taking into consideration the polyphenol and carbohydrate contents and the energy they used, 350 W was considered the fixed power.

#### 2.1.2. Effects of Different Powder Mass on the Carbohydrate and Polyphenol Contents in Artichoke Bud

When the powder mass was in the range of 0.5–1.5 g, the carbohydrate content increased significantly (*p* < 0.05), but when the powder mass continued to increase, the polyphenol and carbohydrate contents always showed an overall decreasing trend (*p* < 0.05) (Figure S1B). It was mainly because, in the aqueous two-phase system, the volume of the upper alcohol phase was small and the volume of the lower water phase was large. When 0.5 g of powder was added, the polyphenols in the upper phase reached a saturated state of dissolution while the carbohydrates did not reach the saturated state. If the mass of the material was increased continuously, other alcohol-soluble components were dissolved and distributed in the upper phase, which affected the extraction of polyphenols, but this had no significant effect on the extraction of carbohydrates in the lower phase [38,39]. A powder mass of 1.0–2.0 g was considered the next factor for response surface optimization taking into consideration the polyphenol and carbohydrate contents.

#### 2.1.3. Effects of Different Extraction Temperatures on Carbohydrate and Polyphenol Contents in Artichoke Bud

The polyphenol and carbohydrate contents showed an increase within the ultrasonic temperature range, and this increasing trend was not altered (*p* < 0.05) (Figure S1C). The study for the extraction of polyphenols and carbohydrates was conducted at 80 °C and not beyond because of the ultrasonic instrument’s maximum allowed temperature limit at 80 °C.

#### 2.1.4. Effects of Different Ultrasonic Times on the Carbohydrate and Polyphenol Contents in Artichoke Bud

The carbohydrate and polyphenol contents showed first an increasing trend and then a decreasing trend with the increase in ultrasonic time and reached their highest levels after 40 min (Figure S1D). This was because, in the early stage of extraction, the cavitation effect of ultrasonic waves promoted the transfer of solutes, accelerated the dissolution of polyphenols and carbohydrates, and increased the content [40]. A continuous increase in the ultrasonic time after the diffusion equilibrium did not cause the dissolution of carbohydrates and polyphenols. Instead, the content was decreased because, with increased ultrasonic power, polyphenols are more easily dissolved, interacting with dissolved carbs to produce insoluble aggregates [36,37]. Therefore, 30–50 min was considered a factor for further response surface optimization.

#### 2.1.5. Effects of Different Concentrations of Ammonium Sulfate on the Carbohydrate and Polyphenol Contents in Artichoke Bud

When the concentration of ammonium sulfate was in the range of 0.27–0.33 g/mL, the carbohydrate content increased significantly (*p* < 0.05), but when the mass concentration of ammonium sulfate continued to increase, the extracted carbohydrate content decreased significantly (*p* < 0.05) (Figure S1E). The variation in the extraction trend of both polyphenols and carbohydrates in this range was roughly the same. The main reason behind it was that the aqueous two-phase system gradually became stable with the continuous increase in the concentration of ammonium sulfate, resulting in a significant increase in the polysaccharide and polyphenol contents. A continuous increase in the concentration of ammonium sulfate increased its solubilization effect, making it easier for polyphenols to distribute in the water phase, which in turn affected the extraction of upper-phase relative polyphenols and lower-phase relative carbohydrates [41]. Hence, the concentration of ammonium sulfate between 0.30 and 0.36 g/mL was considered a factor for response surface optimization.

#### 2.1.6. Effects of Different Alcohol–Water Ratios on the Polysaccharide and Polyphenol Contents in Artichoke Bud

When the alcohol–water ratio was between 0.2 and 0.4, the polysaccharide and polyphenol contents increased significantly (*p* < 0.05), but when the alcohol–water ratio continued to increase, the carbohydrates and polyphenols decreased significantly (*p* < 0.05) (Figure S1F). It was mainly because, with the continuous increase in the volume of ethanol, the aqueous two-phase system was gradually stabilized and the polyphenol and polysaccharide contents increased. However, when the volume of ethanol continued to increase, some other substances (such as proteins) were precipitated, which adsorbed carbohydrates and polyphenols and reduced the polysaccharide and polyphenol contents [42]. Hence, the alcohol–water ratio of 0.3–0.5 was considered a factor for response surface optimization.

### 2.2. Box–Behnken Response Surface Experimental Results

Based on the results of the single-factor experiment, the powder mass (A), ultrasonic time (B), ammonium sulfate concentration (C), and alcohol–water ratio (D) were considered the influencing factors for further response surface optimization, and the polyphenol content (Y_1_) and polysaccharide content (Y_2_) were examined to design a response surface experiment. The response surface factor levels are presented in Table 1, and the experimental scheme and results are provided in Table 2.

The software Design-Expert 11 was used to perform multiple regression analysis and binomial analysis using the data presented in Table 3. The multiple regression equations were: Y_1_ = 5.24 − 0.2067 A + 0.0933 B − 0.0608 C + 0.8308 D + 0.0325 AB − 0.3125 AC + 0.1925 AD − 0.1613 BC − 0.1137 BD + 0.3688 CD − 0.5608 A^2^ − 0.5071 B^2^ − 0.7533 C^2^ − 0.6558 D^2^; Y_2_ = 74.72 − 2.16 A + 1.13 B + 3.33 C − 2.82 D + 0.5938 AB + 2.12 AC + 3.00 AD − 0.6425 BC − 0.3100 BD − 2.37 CD − 7.27 A^2^ − 3.74 B^2^ − 3.22 C^2^ − 5.62 D^2^; the correlation coefficients *r* of Y_1_ and Y_2_ were 0.9772 and 0.9389, respectively, indicating that the independent variable could reflect the change of 97.72% polyphenol content and the change of 93.89% polysaccharide content. The absolute values of the previous coefficients showed how much each factor affected the examined value: the quadratic coefficients of the two models were negative, the parabolic opening of the three-dimensional response surface for each model was downward, and because this could reach the maximum value, we knew that the optimal process could be optimized [43,44].

The results of the analysis of variance are presented in Table 3 and Table 4. The quadratic regression model of polyphenols had strong significance (*p* < 0.0001), the quadratic regression model of carbohydrates also had strong significance (*p* < 0.05), and the lack-of-fit term was not significant (*p* > 0.05), indicating that the two models were well fitted [45]. AC and CD had significant effects on polyphenol content (*p* < 0.05), and D, A^2^, B^2^, C^2^, and D^2^ had extremely significant effects on polyphenol content (*p* < 0.0001) (Table 3). Hence, the effect of polyphenols content was not just an ordinary linear relationship. Table 4 shows that A^2^, B^2^, and D^2^ all significantly affected the carbohydrates content (*p* < 0.05). According to the *p* value, the degree of influence of each factor on Y_1_ was D (alcohol–water ratio) > A (powder mass) > B (ultrasonic time) > C (ammonium sulfate concentration), and the degree of influence on Y_2_ was C (ammonium sulfate concentration) > D (alcohol–water ratio) > A (powder mass) > B (ultrasonic time).

**Figure 1:**
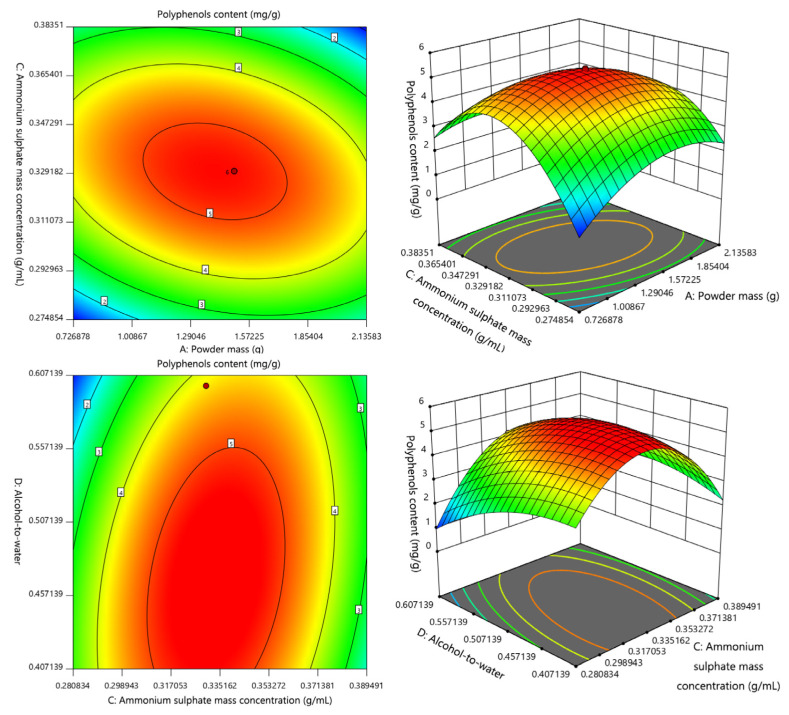
Effect of interaction between the powder mass (A) and the ammonium sulfate concentration (C) or the alcohol–water ratio (D) on the polyphenol content.

The plane contour map and the three-dimensional response surface map are presented in Figure 1, revealing that the interaction of factors had a significant impact on the examined value. The change in the response surface of powder mass was steeper than that of the ammonium sulfate concentration, indicating that the effect of powder mass on polyphenol content was more significant than that of ammonium sulfate concentration. The change in the response surface of the alcohol–water ratio was steeper than that of the ammonium sulfate concentration, indicating that the effect of the alcohol–water ratio on the polyphenol content was more significant than that of the ammonium sulfate concentration. The contour lines on the graph were dense and elliptical in shape, and the corresponding response surface was steep, indicating that the interaction between the powder mass, ammonium sulfate concentration, and alcohol–water ratio had a significant effect on the polyphenol content [46]. The other contour plots and the response surface plots showed that although the overall trend was higher and then lower, the contour lines were sparse, and the response surface was moderate. Hence, the corresponding two factors did not have a significant effect on them. The effect of the two factors on the response values was not significant. The results of this analysis were consistent with the results of the binomial analysis.

The polyphenol content under the optimal process using the ethanol–ammonium sulfate aqueous two-phase system as the solvent in this study compared with the optimization of the extraction process carried out in other studies was significantly higher than those of the other traditional solvents (*p* < 0.05) [35,47]. Furthermore, the polysaccharide content in artichoke bud was significantly higher than that of the polyphenols (*p* < 0.05), which was consistent with the results of Bao et al. [48].

### 2.3. Optimal Process Validation

The optimal process obtained by the response surface software had the powder mass of 1.397 g, ammonium sulfate concentration of 0.335 g/mL, alcohol–water ratio of 0.409, ultrasonic time of 42.60 min, polyphenol content of 5.282 mg/g, and polysaccharide content of 74.845 mg/g. According to the actual situation, the powder mass was 1.4 g, ammonium sulfate concentration was 0.34 g/mL, alcohol–water ratio was 0.4, and ultrasonic time was 43 min. The polyphenol content in artichoke bud was 5.32 ± 0.13 mg/g and the polysaccharide content was 74.78 ± 0.11 mg/g, which were close to the predicted values of the software. Hence, carbohydrates and polyphenols could be effectively taken out of artichoke bud using this improved process.

### 2.4. Evaluation of Antioxidant Activity In Vitro

The higher the free radical scavenging rate, the stronger the antioxidant activity of the sample against the free radicals. Figure S2A,B show that polyphenols reached a scavenging rate of 97.8% at 0.06 mg/mL, while VC at the same concentration reached a scavenging rate of 97.4%. The IC_50_ value of polyphenols substances was 0.01108 mg/mL, and that of standard VC was 0.01294 mg/mL. The IC_50_ values of polyphenols were slightly lower than VC. Therefore, the ability of polyphenols to scavenge ABTS^+^· was extremely strong, which was slightly stronger than that of VC. Carbohydrates reached a scavenging rate of 99.16% at 1.5 mg/mL while VC at the same concentration reached a scavenging rate of 99.86%, which meant that it had a remarkable ability to scavenge ABTS^+^, but it was weaker than that of VC.

The magnitude of the free radical scavenging rate can indicate the strength of the antioxidant activity of the sample against free radicals. Figure S2C,D shows that polyphenols reached a scavenging rate of 70.10% at 0.1 mg/mL while VC at the same concentration reached a scavenging rate of 39.57%. The IC_50_ value of polyphenols was 0.04131 mg/mL, while the IC_50_ value of standard VC was 0.174 mg/mL. The IC_50_ value of polyphenols was significantly lower than that of VC. Therefore, the ability of polyphenols to scavenge DPPH· was strong, which was stronger than that of VC. Carbohydrates reached a scavenging rate of 45.35% at 1.5 mg/mL, while VC at the same concentration reached a scavenging rate of 60.38%, which meant that it had a good ability to scavenge DPPH but it was weaker than that of VC. However, different concentrations of DPPH solutions will lead to different IC_50_ values and low reference-ability. Therefore, we standardized the IC_50_ values of substances inhibiting the DPPH clearance rate according to the methods reported in the references [49], and the standardized results showed that the standardized IC_50_ value of polyphenols was 24.43 ng/nmol, the standardized IC_50_ value of VC was 102.9 ng/nmol, and that of carbohydrates was 1544.35 ng/nmol. These results indicated that the ability of polyphenols to inhibit the DPPH radical was much greater than that of VC, while that of carbohydrates was much less than that of VC.

The level of absorbance can reflect the strength of the reducing power of the sample. The greater the absorbance, the stronger the reducing power of the sample. Figure S2E,F shows that the Fe^3+^-reducing power of polyphenols and carbohydrates and the dose–effect relationship of the concentration of the experimental solution were good. The absorbance of polyphenols at 0.4 mg/mL was 1.3439, while VC at the same concentration reached an absorbance value of 1.2781. The IC_50_ value of polyphenols was 0.06345 mg/mL, and that of standard VC was 0.06976 mg/mL. The IC_50_ value of polyphenols is slightly lower than that of VC. Thus, the ability to reduce Fe^3+^ was extremely strong, which was stronger than that of VC. The absorbance of carbohydrates at 3.5 mg/mL was 1.2672, while VC at the same concentration reached an absorbance value of 3.132. The ability of polyphenols to reduce Fe^3+^ was strong but weaker than that of VC.

The smaller the IC_50_ value of the in vitro antioxidant activity, the stronger the antioxidant activity of the substance and the weaker the antioxidant activity of the substance. Table 5 shows that the antioxidant activity of polyphenols was extremely strong, the ability to scavenge ABTS^+^· was the strongest, and the ability to reduce Fe^3+^ was the weakest. Furthermore, the antioxidant activity of carbohydrates was stronger, and the ability to scavenge ABTS^+^· was the strongest, with the weakest ability to scavenge DPPH. The IC_50_ values of various in vitro antioxidant experiments of polyphenols were lower than those of carbohydrates, indicating that the antioxidant activity of polyphenols was stronger than that of carbohydrates, which was consistent with the findings of Bao et al. [48].

### 2.5. Results of HPLC Analysis of Polysaccharide and Polyphenol Compositions

**Figure 2:**
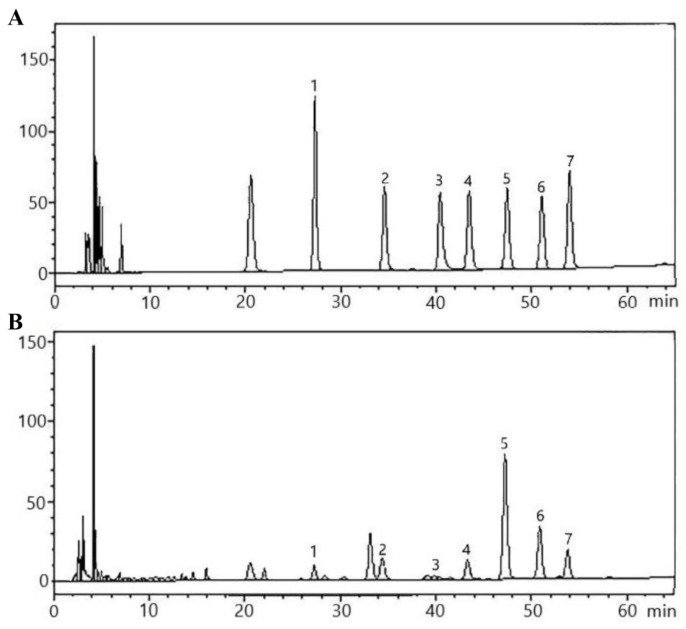
HPLC profile of monosaccharide, alduronic acid mixed standard (**A**) and polysaccharide samples (**B**). 1. Mannose, 2. rhamnose, 3. glucuronic acid, 4. galacturonic acid, 5. glucose, 6. galactose, 7. arabinose.

**Figure 3:**
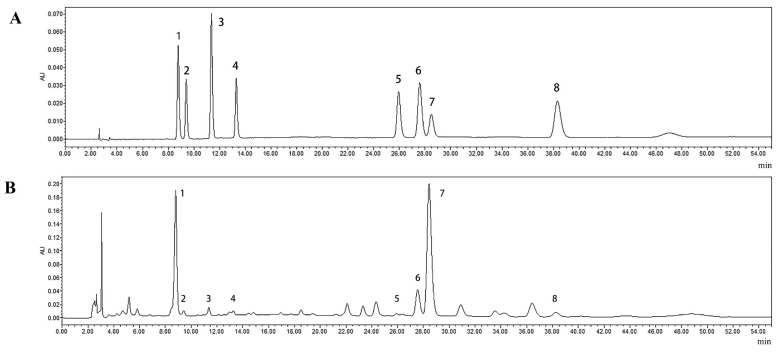
HPLC profile of phenolic acid mixed standard (**A**) and polyphenol samples (**B**). 1. Chlorogenic acid, 2. 4-dicaffeoylquinic acid, 3. caffeic acid, 4. 1,3-dicaffeoylqunic acid, 5. isochlorogenic acid B, 6. isochlorogenic acid A, 7. cynarin, 8. isochlorogenic acid C.

**Figure 4:**
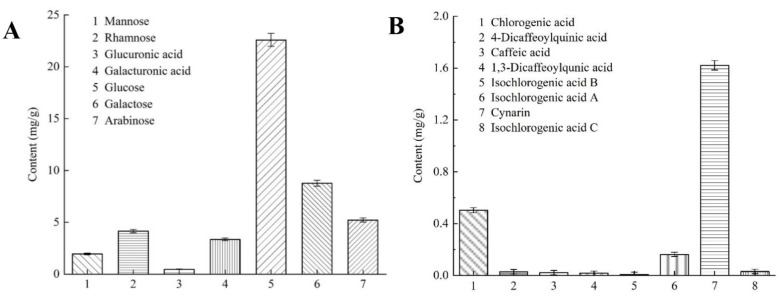
Concentrations of monosaccharides and alduronic acid in carbohydrates (**A**) and concentrations of eight phenolic acids in polyphenols (**B**).

Each monosaccharide, alduronic acid, and phenolic acid standard had a good resolution in the mixed standard, and the peaks of the standard and the sample might be in one-to-one correspondence (Figure 2 and Figure 3). Figure 4A shows the different carbohydrates soluble in ammonium sulfate aqueous solution present in artichoke bud: mannose, rhamnose, glucuronic acid, galacturonic acid, glucose, galactose, and arabinose; the molar ratio was 10.77:25.22:2.37:15.74:125.39:48.62:34.70. Figure 4B shows the constituents of polyphenols present in artichoke bud: chlorogenic acid, 4-dicaffeoylquinic acid, caffeic acid, 1,3-dicaffeoylqunic acid, isochlorogenic acid B, isochlorogenic acid A, cynarin, and isochlorogenic acid C; the contents are 0.503, 0.029, 0.022, 0.017, 0.008, 0.162, 1.621, 0.030 mg/g, respectively. Compared with mass spectrometry, ultraviolet detector has poor sensitivity, so some components with low content may not be detected. With in-depth study, mass spectrometer will be applied to improve the limits of quantification.

As described in Section 2.4, polyphenols and carbohydrates in artichoke bud had strong antioxidant activity. Hence, the combinatory use of carbohydrates and polyphenols as a potential therapeutic alternative for treating relevant disorders with hyperoxidative conditions, such as aging, senescence, etc. Additionally, chlorogenic acid and cynarin were related to the antioxidant activity of artichoke polyphenols [50], but whether other components affected the antioxidant activity of polyphenols is still unknown. Although artichoke carbohydrates had strong antioxidant activity, related studies were few and the components that affected its antioxidant activity needed further exploration. In this study, the HPLC analysis was used to figure out how polyphenols and carbohydrates were made, and it gave us a reference point to learn more about their antioxidant activities.

## 3. Materials and Methods

### 3.1. Materials

Artichoke bud (originating in Yunnan) was obtained from Kunming Mingjian’an Biotechnology Co., Ltd. (Yunnan, China) and identified as dried bud of artichoke (*Cynara scolymus* L.) by Professor Li Jia of Shandong University of Traditional Chinese Medicine. Gallic acid (purity ≥ 98%) was purchased from Shanghai yuanye BioTechnoligy Co., Ltd. (Shanghai, China). Anhydrous Glucose (purity ≥ 99%) was obtained from Shanghai Macklin Biochemical Co., Ltd. (Shanghai, China). Folin phenol reagent, sodium carbonate, phenol, sulfuric acid, ethanol absolute, ammonium sulfate, DPPH, ABTS, potassium persulfate, potassium ferricyanide, trichloroacetic acid, iron trichloride hexahydrate, sodium dihydrogen phosphate, dibasic sodium phosphate, vitamin C (VC), trichloromethane, potassium dihydrogen phosphate, sodium hydroxide, acetic acid, 3-methyl-1-phenyl-2-pyrazolin-5-one, and trifluoroacetic acid are AR grade. Methanol and acetonitrile are HPLC grade. Mannose, Rhamnose, Glucuronic acid, Galacturonic acid, Glucose, Galactose, Arabinose, Chlorogenic acid, 4-Dicaffeoylquinic acid, Caffeic acid, Cynarin, Isochlorogenic acid B, Isochlorogenic acid A, 1,5-Dicaffeoylqunic acid, and Isochlorogenic acid C were purchased from Chengdu Must Biotech Co., LTD (Chengdu, China).

### 3.2. Synchronous Extraction Process

**Figure 5:**
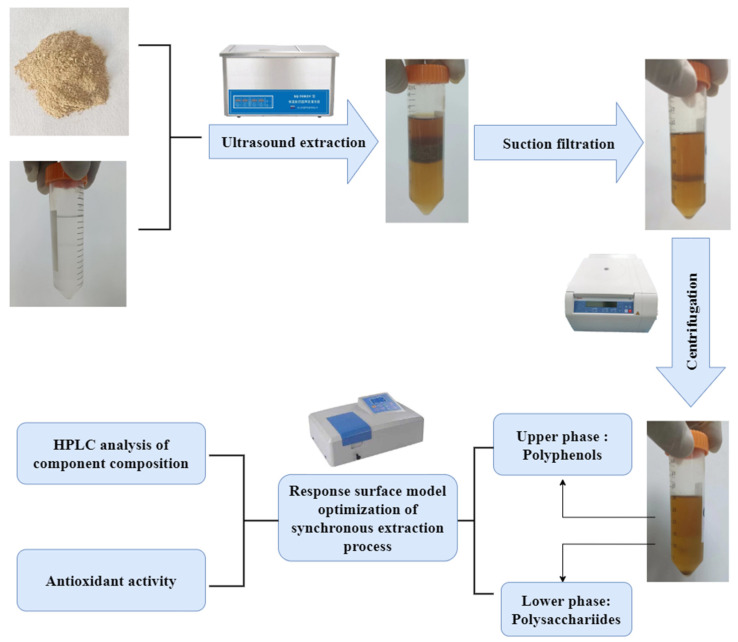
The synchronous extraction process for carbohydrates and polyphenols from artichoke bud.

The synchronous extraction process for carbohydrates and polyphenols from artichoke bud is depicted in Figure 5. The sample solution was prepared as follows: a 50-mL centrifuge tube was filled with 1 g of artichoke bud powder. Then, a 35 mL ethanol-ammonium sulfate two-phase system was prepared by adding ammonium sulfate with a mass concentration of 0.33 g/mL and an alcohol–water ratio of 0.4. The extraction was carried out under fixed conditions of ultrasonic power of 500 W, an ultrasonic time of 30 min and an ultrasonic temperature of 60 °C in the ultrasonic cleaner. Suction filtration and centrifugation were at 7000 rpm for 10 min.

### 3.3. Determination of Carbohydrate Content

The phenol–sulfuric acid method was used with minor modifications to determine the carbohydrate content [51]. The precisely pipetted 1.0 mL of the lower-phase solution diluted 50 times, 1.0 mL of distilled water, and 1.0 mL of a 5% phenol solution were mixed well, making up to 8.0 mL with concentrated sulfuric acid. The mixture was then left for 10 min at room temperature, placed in a water bath at 40 °C for 15 min, and cooled at room temperature. The absorbance was measured at 490 nm, and the average value was taken in triplicate. Anhydrous glucose was used as a standard and the results were expressed as milligrams of anhydrous glucose equivalents per gram artichoke bud sample.

### 3.4. Determination of Polyphenol Content

The polyphenol content was determined using the Folin–Ciocalteu colorimetric method [34]. The precisely pipetted 0.5 mL of the upper-phase solution and 2.0 mL of the Folin–Ciocalteu reagent with a volume fraction of 50% were mixed well. The mixture was left at room temperature for 5 min, and 4.0 mL of 0.2 g/L Na_2_CO_3_ solution was added to the mixture, making up to 10 mL with distilled water. The mixture was kept away from light at room temperature for 2 h. The absorbance was measured at 760 nm, and the average value was taken in triplicate. Gallic acid was used as a standard and the results were expressed as milligrams of gallic acid equivalents per gram artichoke bud sample.

### 3.5. Single-Factor Experiment

The effects of various factors on carbohydrates and polyphenols were examined using on ultrasonic power (300, 350, 400, 450, and 500 W), powder mass (0.5, 1.0, 1.5, 2.0, and 2.5 g), extraction temperature (40, 50, 60, 70, and 80 °C), ultrasonic time (20, 30, 40, 50, and 60 min), ammonium sulfate mass concentration (0.27, 0.30, 0.33, 0.36, and 0.39 g/mL), and alcohol–water ratio (0.2, 0.3, 0.4, 0.5, and 0.6).

### 3.6. Response Surface Experiment

A response surface experiment was performed for optimization based on the high and low levels in the single-factor experiment using carbohydrates and polyphenol contents as variables.

### 3.7. Determination of Antioxidant Activity In Vitro

#### 3.7.1. Determination of ABTS^+^· Scavenging Capacity

Referring to the method in the literature [52], we used 0.3 mL of specific concentration of lower-phase solution and upper-phase solution of 0.3 mL was aspirated to measure the ABTS^+^ scavenging capacity. The curves depicting the scavenging rates of carbohydrates and polyphenols to ABTS^+^· were drawn, and the IC_50_ value was calculated. The same concentration of VC was used as a positive control.

#### 3.7.2. Determination of DPPH· Scavenging Capacity

We followed the method available in the literature with minor modifications to determine the scavenging capacity of DPPH [53]. For this, 0.3 mL of a specific concentration of the lower-phase solution and upper-phase solution was aspirated and mixed with 2.0 mL of DPPH· solution (0.1 mg/mL), making up to 10 mL with distilled water. The reaction was performed at room temperature for 30 min in the dark. The absorbance *A*_1_ was measured at 517 nm. Next, 0.3 mL of the lower-phase solution and upper-phase solution was mixed with 2.0 mL of absolute ethanol, making up to 10 mL with distilled water. The reaction was performed at room temperature for 30 min in the dark and then the absorbance *A*_2_ was measured at 517 nm. The curves depicting the scavenging rates of carbohydrates and polyphenols to DPPH· were drawn and IC_50_ values were calculated. The same concentration of VC was used as a positive control.

$$\mathrm{DPPH}\cdot \;\mathrm{scavenging}\;\mathrm{rate}\;\left(\%\right)=\left(1-\frac{A_{1}-A_{2}}{A_{0}}\right)\times 100\%$$

#### 3.7.3. Determination of Fe^3+^-Reducing Power

The method available in the literature was followed with minor modifications to determine the reducing capacity of Fe^3+^ [54]. Further, 0.3 mL of the lower-phase solution or upper-phase solution, 2.5 mL of phosphate buffer solution (pH = 7.4), and 2.5 mL of 1% K_3_ [Fe(CN)_6_] solution were mixed. The mixture was then placed in a water bath for 30 min at 50 °C. It was taken out and cooled to room temperature. Further, 1.0 mL of TCA was added, and the mixture was allowed to stand for 20 min before adding 2.5 mL of the supernatant, 0.5 mL of 0.1% FeCl_3_, and 2.5 mL of distilled water to it. The mixture was mixed well and left for 10 min. The absorbance was measured at 700 nm. The curves depicting Abs of carbohydrates and polyphenols were drawn, and IC_50_ values were calculated. The same concentration of VC was used as a positive control.

### 3.8. High-Performance Liquid Chromatography Analysis

#### 3.8.1. Standard Solution Preparation

Appropriate masses of monosaccharides and uronic acid standards were accurately weighed and dissolved to prepare a 1 mg/mL mixed standard solution.

An appropriate mass of each phenolic acid standard was accurately weighed and dissolved to prepare a 0.2 mg/mL mixed standard solution.

#### 3.8.2. Sample Solution Preparation

The lower-phase carbohydrate stock solution was freeze-dried as described in Section 3.2, and an appropriate mass of freeze-dried powder was weighed and dissolved to prepare the carbohydrate solution. To obtain the mixed standard solution and sample solution, 2 mL of 4 mol/L TFA solution was added to the carbohydrate solution and mixed well. The mixture was then hydrolyzed in an oven at 110 °C for 2 h, taken out, cooled to room temperature, and adjusted to neutral with 4 mol/L NaOH, making up to 5 mL with distilled water. We then took 200 μL of this hydrolyzed solution and added 200 μL of 0.3 mol/L NaOH solution and 200 μL of 0.5 mol/L PMP methanol solution and mixed the solution well. The solution was then placed in a water bath for 70 min at 70 °C. The solution was allowed to cool to room temperature. The pH of the solution was then adjusted by adding 200 μL of a 0.3 mol/L acetic acid solution and 200 μL of a 0.1 mol/L KH_2_PO_4_ (pH = 6.0) solution. We then added 2 mL of CHCl_4_ and vortexed the solution well to remove the chloroform phase. It was done thrice. The chromatographic analysis was performed on the solution after passing it through a 0.45-μm aqueous film.

The polyphenol solution was obtained using an appropriate volume of the upper-phase stock solution prepared as described in Section 3.2.

#### 3.8.3. Chromatographic Conditions

The high-performance liquid chromatography (HPLC) conditions of carbohydrates and polyphenols are listed in Supplementary Table S1.

### 3.9. Statistical Analyses

Origin Pro 8 (OriginLab Corporation, Northampton, MA, USA) was used to analyze and map the experimental data, and Design-Expert 11 (Stat-Ease, Inc., Minneapolis, MN, USA) was used to design and analyze response surface experiments. IC_50_ calculations were performed using Origin Pro 8 software.

## 4. Conclusions

In this study, the powder mass, ammonium sulfate concentration, alcohol–water ratio, and ultrasonic time were considered the optimizing factors, and the optimum process conditions were obtained by constructing a double-response surface model: powder mass of 1.4 g, ammonium sulfate concentration of 0.34 g/mL, alcohol–water ratio of 0.4, and ultrasonic time of 43 min. Under these conditions, the polyphenol content in artichoke bud was 5.32 ± 0.13 mg/g and the polysaccharide content was 74.78 ± 0.11 mg/g. The HPLC spectrum analysis revealed that the carbohydrates in artichoke bud comprised mannose, rhamnose, glucuronic acid, galacturonic acid, glucose, galactose, and arabinose, with a molar ratio of 10.77:25.22:2.37:15.74:125.39:48.62:34.70. The polyphenols comprised chlorogenic acid, 4-dicaffeoylquinic acid, caffeic acid, 1,3-dicaffeoylqunic acid, isochlorogenic acid B, isochlorogenic acid A, cynarin, and isochlorogenic acid C, and the contents were 0.503, 0.029, 0.022, 0.017, 0.008, 0.162, 1.621, 0.030 mg/g, respectively. The antioxidant activity of ABTS^+^· and DPPH· scavenging and Fe^3+^-reducing abilities was determined in vitro. The results showed that the antioxidant activity of carbohydrates and polyphenols in artichoke bud was strong, and the antioxidant activity of polyphenols was stronger than those of carbohydrates.

In an aqueous two-phase system, the combination of ethanol and saline solution can be considered an environmentally friendly method for extracting biologically active substances. These mixtures are not only low-cost and simple to prepare but also provide a mild and nontoxic environment. In addition, replacing traditional solvents with an aqueous two-phase system has shown great benefits in extracting natural plant components, such as shorter extraction times and higher extraction efficiencies. Its use in combination with ultrasonic power provides a rapid and effective method to extract polyphenols and carbohydrates from artichoke bud. The optimal extraction process obtained by optimizing the dual-response surface model was stable and feasible, the carbohydrates and polyphenols obtained by crude extraction had good antioxidative effects in vitro, and the material composition was obtained by HPLC analysis. These advantages provided a theoretical basis for exploring the biological activities of carbohydrates and polyphenols, and for resource development and utilization in more detail.

## Figures and Tables

**Table 1:** Response surface factor levels.

Levels	Factors			
	A Powder Mass (g)	B Ultrasound Time (min)	C Ammonium Sulphate Mass Concentration (g/mL)	D Alcohol–Water Ratio
−1	1.0	30	0.30	0.3
0	1.5	40	0.33	0.4
1	2.0	50	0.36	0.5

**Table 2:** Response Surface Experiment Scheme and Results.

No.	A	B	C	D	The Content of Polyphenols (mg/g)	The Content of Carbohydrates (mg/g)
1	1	30	0.3	0.3	2.17	56.61
2	2	30	0.3	0.3	1.56	26.60
3	1	50	0.3	0.3	3.14	52.37
4	2	50	0.3	0.3	2.68	50.80
5	1	30	0.36	0.3	1.64	57.57
6	2	30	0.36	0.3	1.45	63.11
7	1	50	0.36	0.3	1.82	67.23
8	2	50	0.36	0.3	1.18	56.64
9	1	30	0.3	0.5	2.56	46.12
10	2	30	0.3	0.5	3.91	46.85
11	1	50	0.3	0.5	2.45	48.81
12	2	50	0.3	0.5	4.31	50.01
13	1	30	0.36	0.5	4.53	46.76
14	2	30	0.36	0.5	3.36	53.08
15	1	50	0.36	0.5	4.32	54.57
16	2	50	0.36	0.5	3.46	57.61
17	0.5	40	0.33	0.4	4.00	57.69
18	2.5	40	0.33	0.4	1.88	44.41
19	1.5	20	0.33	0.4	3.14	68.72
20	1.5	60	0.33	0.4	3.17	61.63
21	1.5	40	0.27	0.4	2.28	66.85
22	1.5	40	0.39	0.4	2.06	67.61
23	1.5	40	0.33	0.2	0.89	67.77
24	1.5	40	0.33	0.6	4.23	47.53
25	1.5	40	0.33	0.4	5.21	74.77
26	1.5	40	0.33	0.4	5.22	74.69
27	1.5	40	0.33	0.4	5.23	74.72
28	1.5	40	0.33	0.4	5.28	74.76
29	1.5	40	0.33	0.4	5.23	74.68
30	1.5	40	0.33	0.4	5.24	74.72

**Table 3:** Analysis of variance for polyphenols content.

Source	Sum of Squares of Deviation from Mean	Degrees of Freedom	Mean Square	*F* Value	*p* Value
Model	53.44	14	3.82	15.99	<0.0001
A	1.03	1	1.03	4.29	0.0559
B	0.2091	1	0.2091	0.8755	0.3643
C	0.0888	1	0.0888	0.3719	0.5511
D	16.57	1	16.57	69.38	0.0001
AB	0.0169	1	0.0169	0.0708	0.7938
AC	1.56	1	1.56	6.54	0.0218
AD	0.5929	1	0.5929	2.48	0.1359
BC	0.4160	1	0.4160	1.74	0.2066
BD	0.2070	1	0.2070	0.8670	0.3665
CD	2.18	1	2.18	9.11	0.0086
A^2^	8.63	1	8.63	36.13	<0.0001
B^2^	7.05	1	7.05	29.54	<0.0001
C^2^	15.57	1	15.57	65.19	<0.0001
D^2^	11.80	1	11.80	49.41	<0.0001
Residual	3.58	15	0.2388	—	—
Lack of fit	1.29	10	0.1290	4.23	0.1969
Pure Error	0.0030	5	0.0006	—	—
Sum error	57.03	29	—	—	—

**Table 4:** Analysis of variance for carbohydrates content.

Source	Sum of Squares of Deviation from Mean	Degrees of Freedom	Mean Square	*F* Value	*p* Value
Model	3138.99	14	224.21	3.48	0.0112
A	112.23	1	112.23	1.74	0.2069
B	30.74	1	30.74	0.4766	0.5005
C	266.13	1	266.13	4.13	0.0603
D	190.41	1	190.41	2.95	0.1063
AB	5.64	1	5.64	0.0875	0.7715
AC	72.08	1	72.08	1.12	0.3071
AD	143.52	1	143.52	2.23	0.1565
BC	6.60	1	6.60	0.1024	0.7534
BD	1.54	1	1.54	0.0238	0.8793
CD	89.97	1	89.97	1.40	0.2559
A^2^	1449.35	1	1449.35	22.47	0.0003
B^2^	383.23	1	383.23	5.94	0.0277
C^2^	285.13	1	285.13	4.42	0.0528
D^2^	866.06	1	866.06	13.43	0.0023
Residual	967.32	15	64.49	—	—
Lack of fit	1.56	10	0.1560	1.62	0.3191
Pure Error	0.0065	5	0.0013	—	—
Sum error	4106.32	29	—	—	—

**Table 5:** In vitro antioxidant experimental IC_50_ values.

Experiment Name	Carbohydrates	Polyphenols	VC
Clear ABTS^+^	0.4141 mg/mL	0.01108 mg/mL	0.01294 mg/mL
Clear DPPH	2.6110 mg/mL	0.04131 mg/mL	0.17400 mg/mL
Fe^3+^ reducing power	0.9363 mg/mL	0.06345 mg/mL	0.06976 mg/mL

## Data Availability

The data are contained within the article.

## Supplementary Materials

The following supporting information can be downloaded at: https://www.mdpi.com/article/10.3390/molecules27248962/s1, Table S1 HPLC conditions of carbohydrates and polyphenols; Figure S1 Influence of ultrasonic power (A), powder mass (B), extraction temperature (C), ultrasonic time (D), mass concentration of ammonium sulphate (E), and alcohol-to-water ratio (F) on the extraction of carbohydrates and polyphenols from artichoke bud; Figure S2 In vitro antioxidant experimental evaluation of carbohydrates and polyphenols.

## Sample Availability

Samples of the compounds chlorogenic acid, caffeic acid, isochlorogenic acid B, isochlorogenic acid A, cynarin, and isochlorogenic acid C are available from the authors.
