# Supplementary material for: Synchronous Extraction, Antioxidant Activity Evaluation, and Composition Analysis of Carbohydrates and Polyphenols Present in Artichoke Bud
Source: Molecules. 2022 Dec 16;27(24):8962. doi: 10.3390/molecules27248962 (PMC9783601; doi:10.3390/molecules27248962)
Supplement: Supplementary file 1 [file molecules-27-08962-s001.zip › molecules-2077926-supplementary.pdf]

Outline of the supplementary material

Tables

Table S1 HPLC conditions of carbohydrates and polyphenols.

Figures

Fig. S1 Influence of ultrasonic power (A), powder mass (B), extraction temperature (C), ultrasonic time (D), mass concentration of ammonium sulphate (E), and alcohol-to-water ratio (F) on the extraction of carbohydrates and polyphenols from artichoke bud.

Fig. S2 *In vitro* antioxidant experimental evaluation of carbohydrates and polyphenols.

Supplementary Table S1 HPLC conditions of carbohydrates and polyphenols.

Condition	carbohydrates	Polyphenols
Chromatographic columns	Thermo Scientific™ Hypersil GOLD C_{18} (ThermoFisher Scientific, MA, USA) (250 × 4.6 mm^2, 5 μm)	Agilent ZORBAX SB-C_{18} (Agilent Technologies, CA, USA) (250 × 4.6 mm^2, 5 μm)
Mobile phase	A: 15% acetonitrile (*v*/*v*)<br>B: 50 mmol/L phosphate buffer (KH_2PO_4–NaOH, pH = 6.0)	A: methanol–acetonitrile (1:1)<br>B: 0.1% phosphoric acid solution
Injection volume (μL)	20	10
Temperature (℃)	30	30
Detection wavelength (nm)	250	327
Flow rate (mL/min)	1.0	1.0
Time required for gradient elution	0 → 9→35→ 45→ 55 min	0→ 15→ 45 →60 min
Concentration of gradient elution (mobile phase A)	100%→92%→80% →75% →100%	15 % → 25 % →25 % →30 %

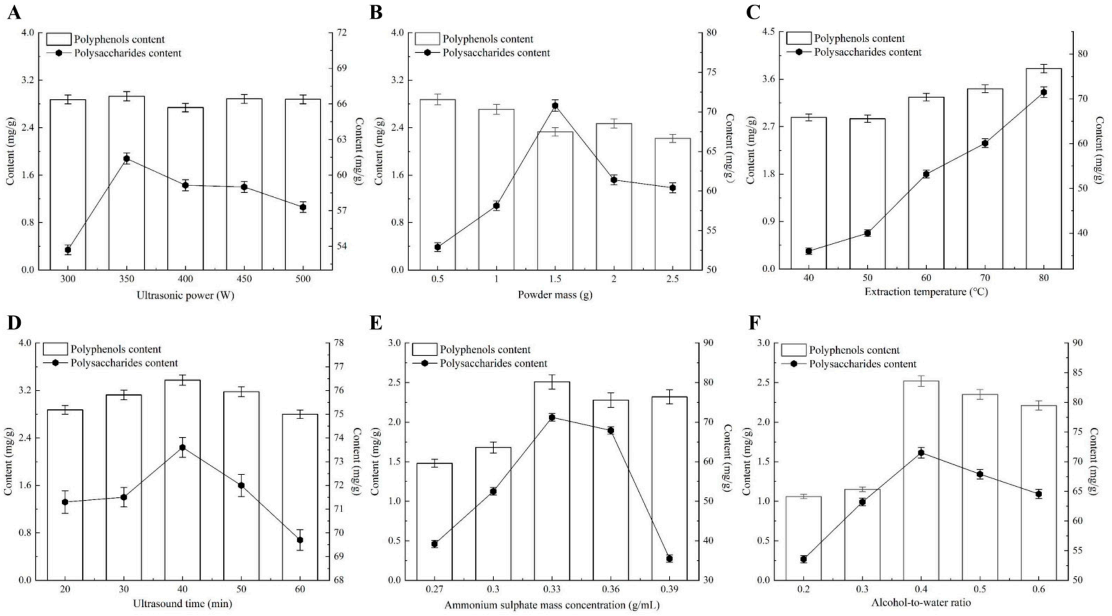

Supplemental Fig. S1 Influence of ultrasonic power (A), powder mass (B), extraction temperature (C), ultrasonic time (D), mass concentration of ammonium sulphate (E), and alcohol-to-water ratio (F) on the extraction of carbohydrates and polyphenols from artichoke bud.

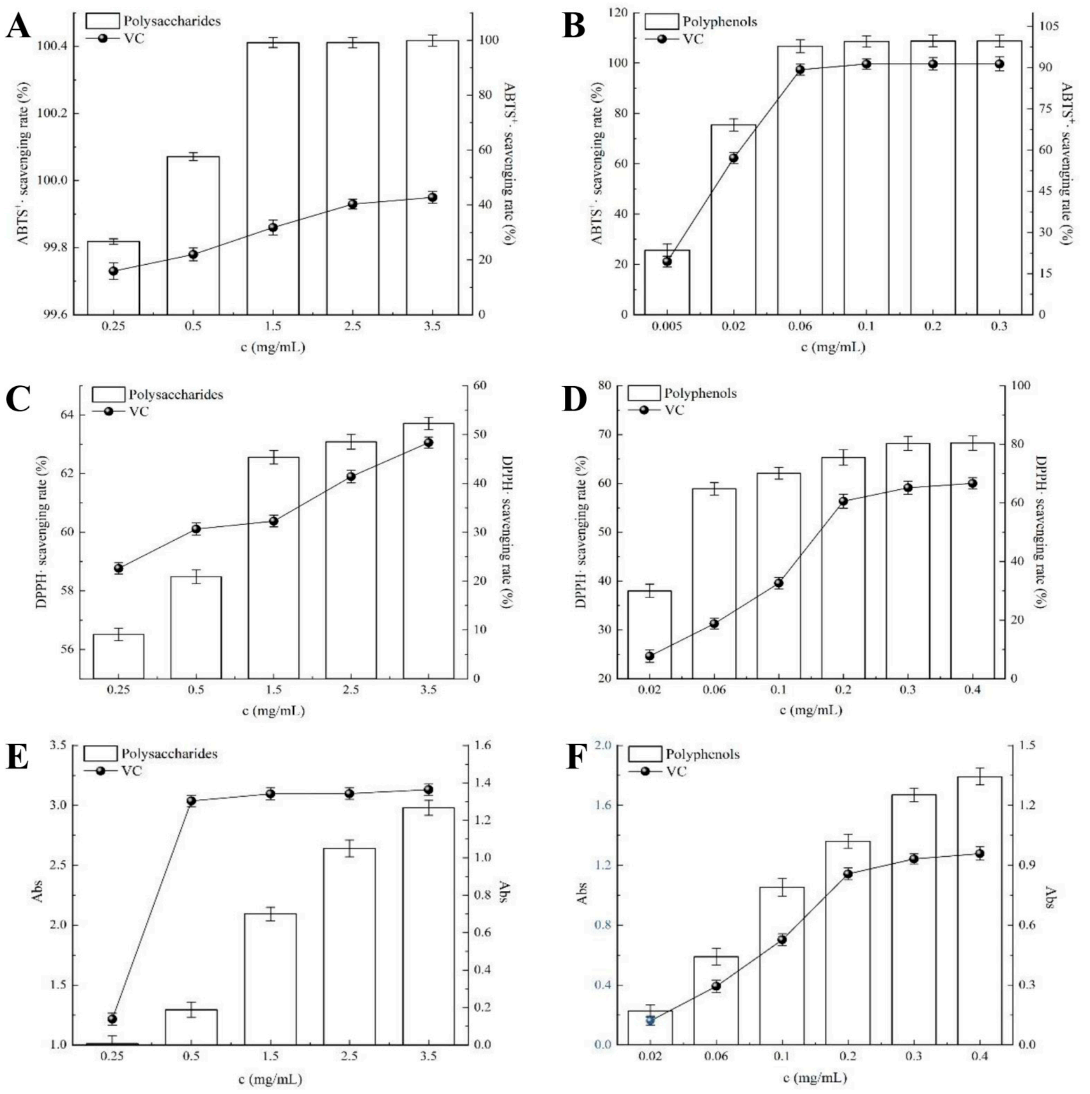

Supplemental Fig. S2 *In vitro* antioxidant experimental evaluation of carbohydrates and polyphenols.
